# A combination of bimalleolar fracture and fracture on talar body and neck: A rare case report

**DOI:** 10.1097/MD.0000000000020862

**Published:** 2020-06-26

**Authors:** Kuan-Ju Chen, Chih-Yuan Ko, Tsung-Yu Ho, Hsien-Te Chen, Horng-Chaung Hsu, Chih-Hung Hung

**Affiliations:** a Department of Orthopedic Surgery, China Medical University Hospital; b Spine Center, China Medical University Hospital; c Department of Sport Medicine, College of Health Care; d Department of Orthopedic Surgery, School of Medicine, China Medical University, Taichung, Taiwan, R.O.C.

**Keywords:** ankle fracture, talar fracture, malleolar fracture, surgery for ankle fracture, talus

## Abstract

**Rationale:**

Talar fracture accompanied with malleolar fracture is rare, and its management is complex. Ankle soft tissue is much thinner than other parts of the human body, and the shape of the ankle makes wounds difficult to close immediately after surgery, which may result in poor skin condition if the wound tension is too high. However, joint congruity and osteonecrosis are the main concerns of talar fracture.

**Patient concerns:**

A 57-year-old man presented at the emergency department following a motorcycle accident.

**Diagnoses:**

Physical examination revealed swelling and tenderness of the left ankle and midfoot. The patient had comminuted talar fracture and was indicated for dual-screw fixation or even plate with screw fixation.

**Interventions:**

We performed single screw fixation after assessing the soft tissue condition and employed a technique of using continuous longitudinal force to bring together fracture fragments (ankle ligamentotaxis) during surgery. Open reduction with a mini-hook plate and tension band wire was used for bimalleolar fracture repair using the combined anteromedial and anterolateral approach with extension of the incision. Kirschner wire for temporary fixation was performed using ligamentotaxis, and a 2.4 headless screw was inserted from the posteromedial to the anterolateral direction.

**Outcomes:**

The patient was discharged with a standard short leg splint and was instructed not to bear weight on the affected ankle for 2 months. The patient walked well without discomfort, and the Hawkins sign was clearly visible. Single screw fixation preserves the integrity of the talus bone as minimal space is used for this operative technique. Single screw fixation preserves more bony stock when most of the internal fixator is located within the bone. Additionally, surgery time is shorter than multiple implantations even when performing the same procedure; as a result, there was less ankle soft tissue swelling.

**Lessons:**

This case provides evidence of using the single screw fixation technique for addressing both malleolar and talar fractures, and that talar fracture management can be less aggressive with limited weight bearing and initial limited range of motion given the presence of malleolar fracture. The alignment and stability of bony fragments also benefit from ankle ligamentotaxis.

## 1 Introduction

Talar fractures are commonly caused by vertical force and are rarely concurrent with malleolar fractures. Only a few cases of concomitant talar and malleolar fractures exist in the literature,^[1–3]^ which accounts for 0.3% of bone fractures and 3.4% of foot fractures.^[2]^ In patients with displaced talus fractures, 90% develop post-traumatic hindfoot arthrosis.^[4]^ Injuries associated with medial malleolar fracture are less likely to develop avascular necrosis (AVN).^[2]^ Titanium screws are commonly used in fracture repair and compatible with magnetic resonance imaging, which can detect AVN shortly after surgery.^[4]^ Talar neck fractures are usually caused by vertical compression through the calcaneus, forcing the talus against the anterior tibia.^[5]^

We present an unusual case of a sagittal plane fracture of the talar neck and body combined with a bimalleolar fracture. The patient was treated with open reduction and internal fixation with a mini-hook and tension band wire for the bimalleolar fracture and a single headless screw for the talus fracture.

## 2 Case report

A 57-year-old man presented at the emergency department following a motorcycle accident with unclear injury mechanism and speed. Physical examination revealed swelling and tenderness of the left shoulder, left upper chest, left ankle, and midfoot. After radiography and computed tomography, he was diagnosed with fractures of the left clavicle, left ribs, left malleoli, left talus, cuboid bone, anterior process of the calcaneus, and navicular bone. The malleolar fractures were classified as open I and supination-adduction stage II based on the Gustilo and Lauge–Hansen classification systems, respectively. The talar fracture was classified as AO Foundation and Orthopedic Trauma Association classification: 81.1.B1, 81.1.C3, and 81.2.A. It was also classified as Hawkin type I with neck communication.^[6,7]^

**Figure 1:**
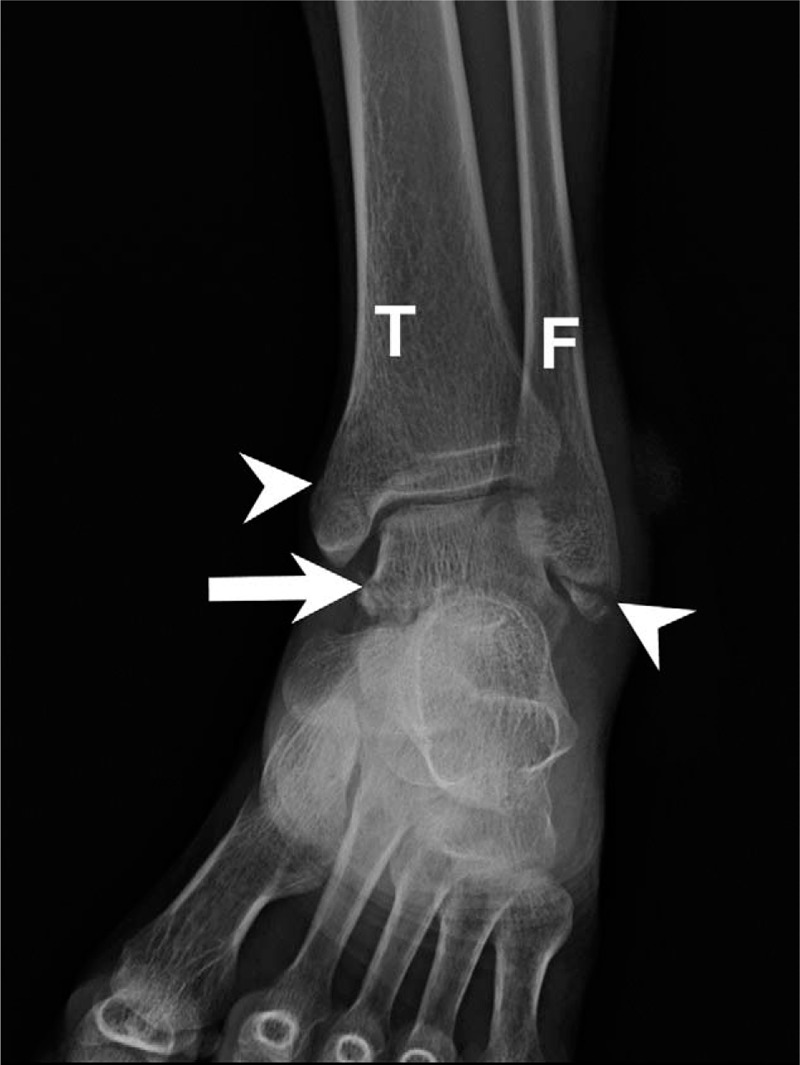
Fractures resulting from an accident in a 57-yr-old patient. Talar fracture with bimalleolar fracture diagnosed on an ankle anteroposterior plain film. Arrow = talus, Arrowhead = malleolar fracture site; F = fibula; T = tibia.

**Figure 2:**
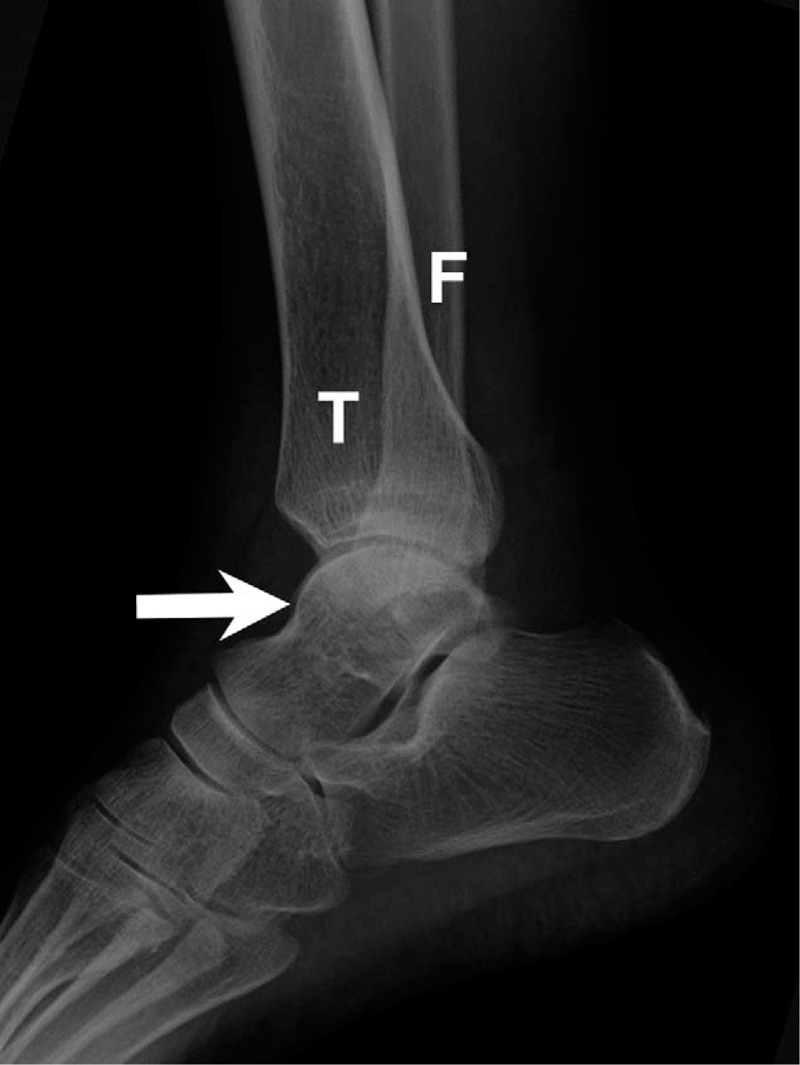
Talar fracture with bimalleolar fracture. Ankle lateral plain film. Arrow = talus, F = fibula, T = tibia.

**Figure 3:**
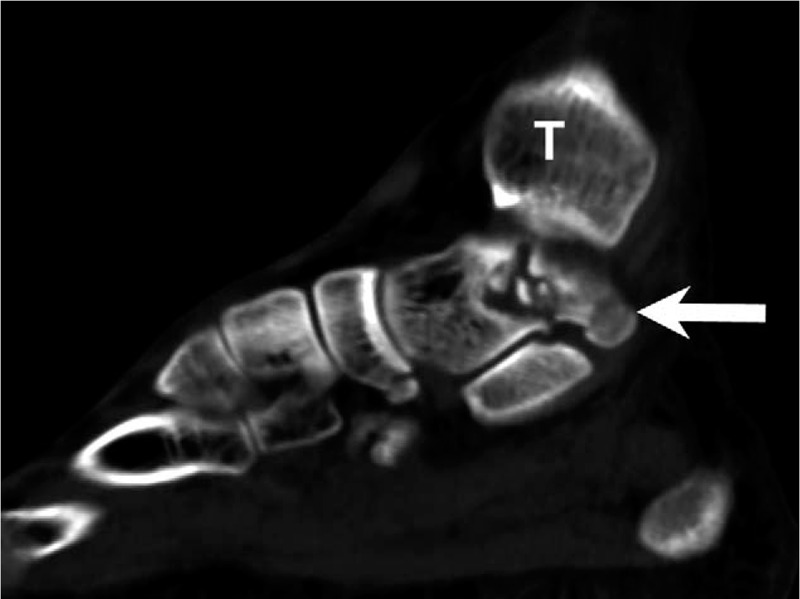
In congruity examination, computed tomography was used to diagnose talar neck fracture. Arrow = talus, T = tibia.

**Figure 4:**
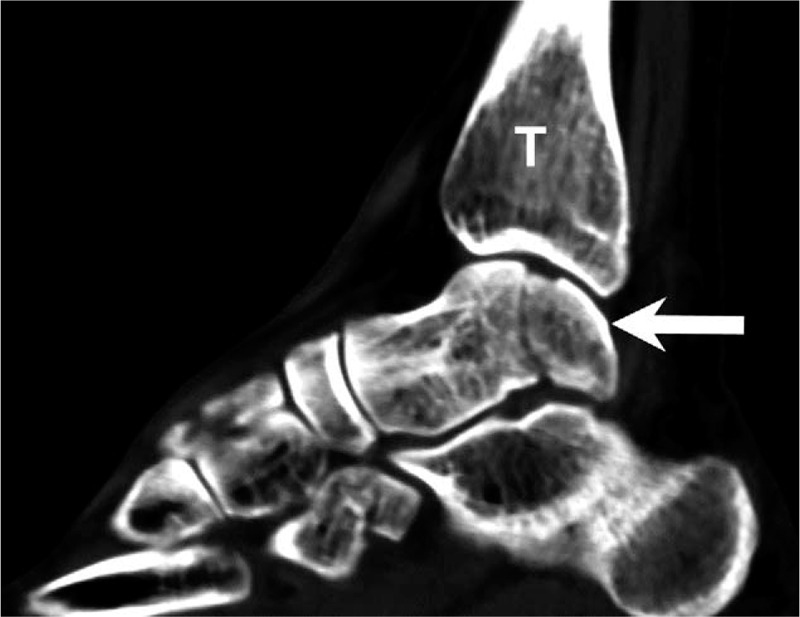
Computed tomography image of the talar body fracture. Arrow = talus, T = tibia.

**Figure 5:**
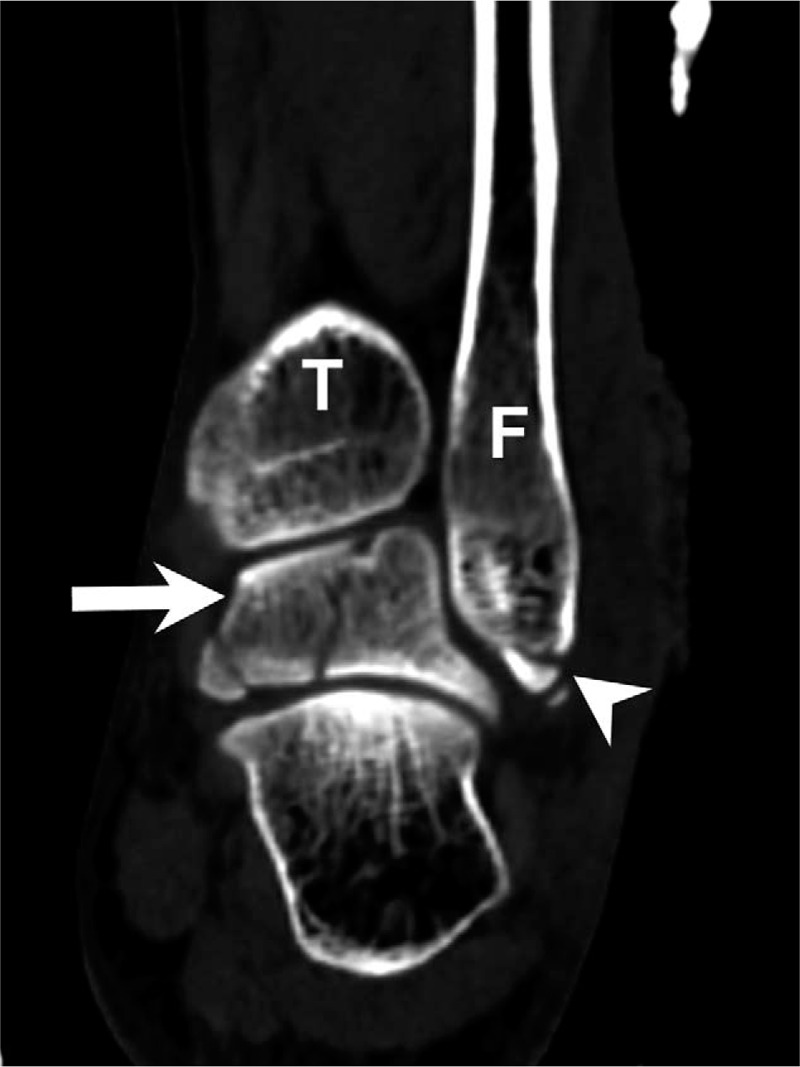
Computed tomography coronary view of the talar fracture with 3 main fragments. Arrowhead = lateral malleolar fracture site, Arrow = talus, F = fibula, T = tibia.

**Figure 6:**
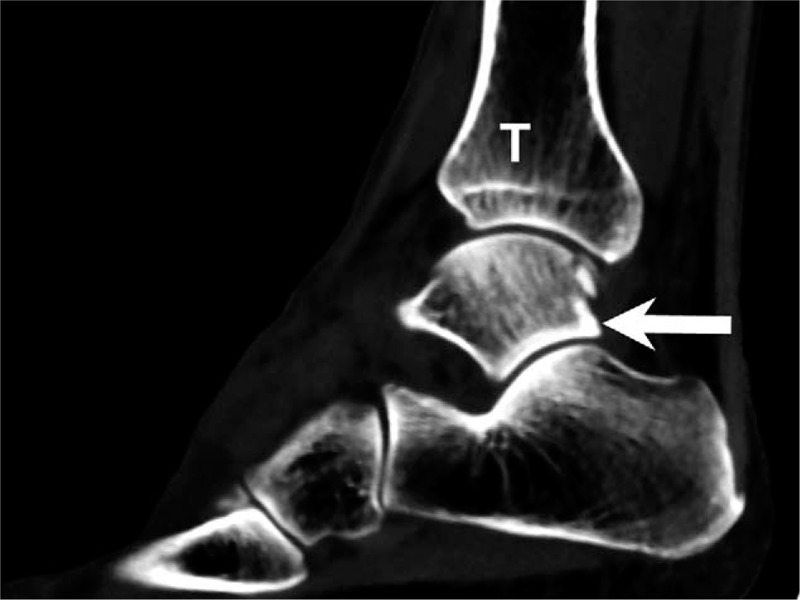
Computed tomography sagittal view of the posterior tubercle fracture of the talus. Arrow = talus, F = fibula, T = tibia.

The initial plain film (Figs. 1 and 2) showed a bimalleolar fracture with a small fracture line over the medial talus. Figure 3 shows the comminution of the talar neck fracture. Figures 4 and 5 show the talar body fracture. Figure 6 shows the talar posterior tubercle fracture.

The patient agreed to the publication of this case report and provided a signed informed consent form.

## 3 Treatment

**Figure 7:**
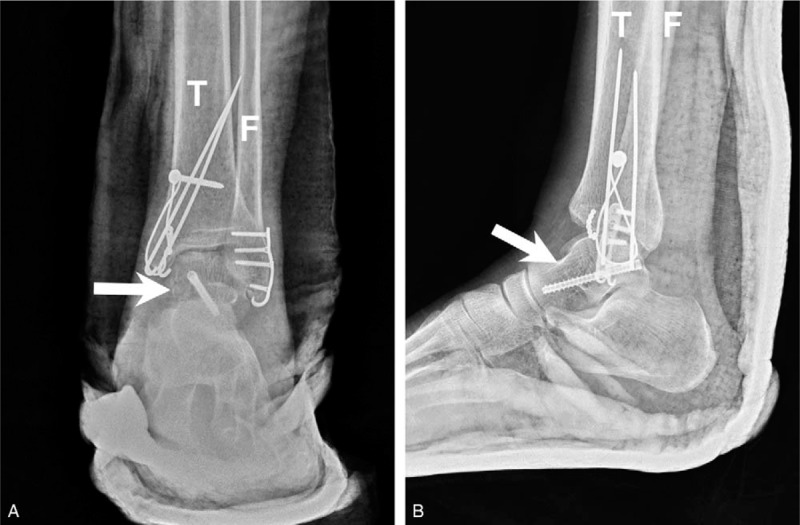
Plain films of the ankle after surgery: anteroposterior and lateral views. Note **single screw** fixation of the talar fracture. Arrow = talus, T = tibia.

**Figure 8:**
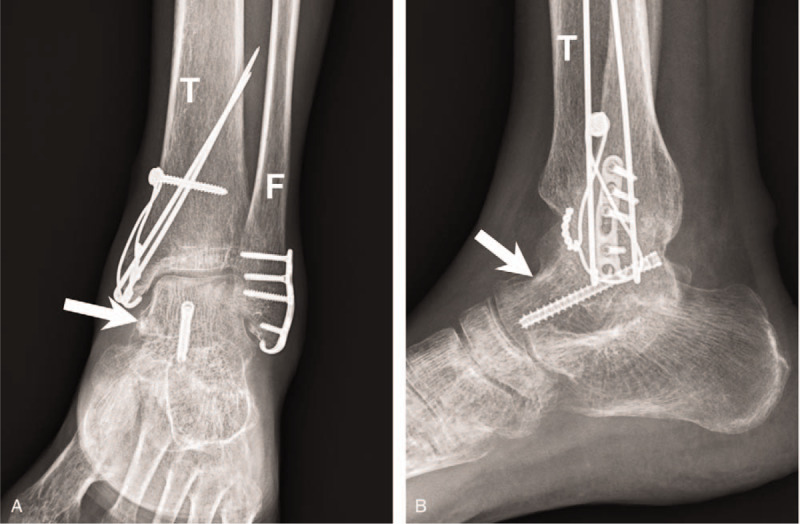
Surgery site 3 mo after surgery. Hawkins sign is noted on anteroposterior and lateral views. Arrow = talus, F = fibula, T = tibia.

**Figure 9:**
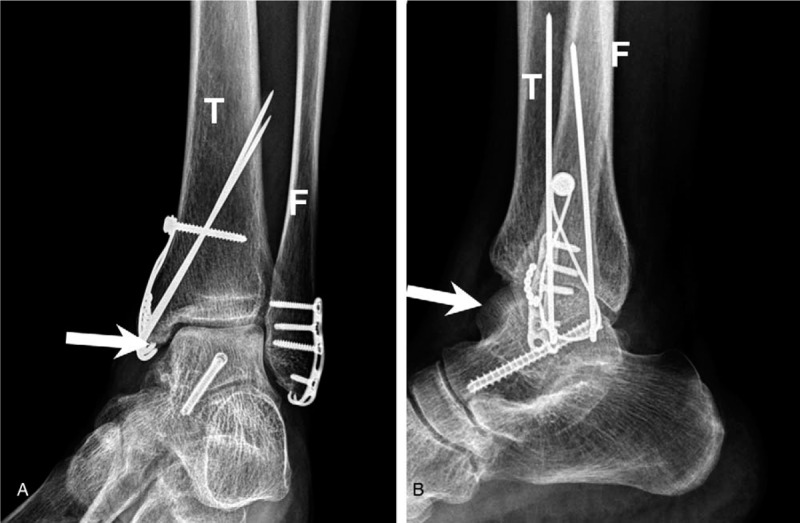
Surgery site 10 mon after surgery. Hawkins sign is clear on the anteroposterior and lateral views. Arrow = talus, F = fibula, T = tibia.

Open reduction with a mini-hook plate and tension band wire was used for bimalleolar fracture repair using the combined anteromedial and anterolateral approach with extension of the incision. Although the computed tomography revealed that the talus was comminuted over the neck portion, step-off of talar surface was less than 2 mm under fluoroscopy and congruity was confirmed by Kirschner wire directly swept. In this method, we avoided excessive disruption of ligaments and also ensured the reduction of talus fragment. Kirschner wire for temporary fixation was performed using ligamentotaxis, and a 2.4 headless screw was inserted from the posteromedial to the anterolateral direction. Ankle range of motion and stability of the bony fragment were analyzed using real-time fluoroscopy during surgery. Operating time is 3 hours. Figure 7 shows the postoperative anteroposterior + lateral plain films. The patient was discharged with a standard short leg splint and was instructed not to bear weight on the affected ankle for 2 months. Figure 8 was obtained 3 months after surgery, and the patient was able to walk at that time. Figure 9 shows the 10-month postoperative results. The patient walked well without discomfort, and the Hawkins sign was clearly visible.

On the last follow up, the patient scored 90 on the Baird and Jackson Scoring System^[8]^ (Table 1) and 95 on the American orthopedic foot and ankle society ankle-hindfoot scale^[9]^ (Table 2).

**Table 1:**
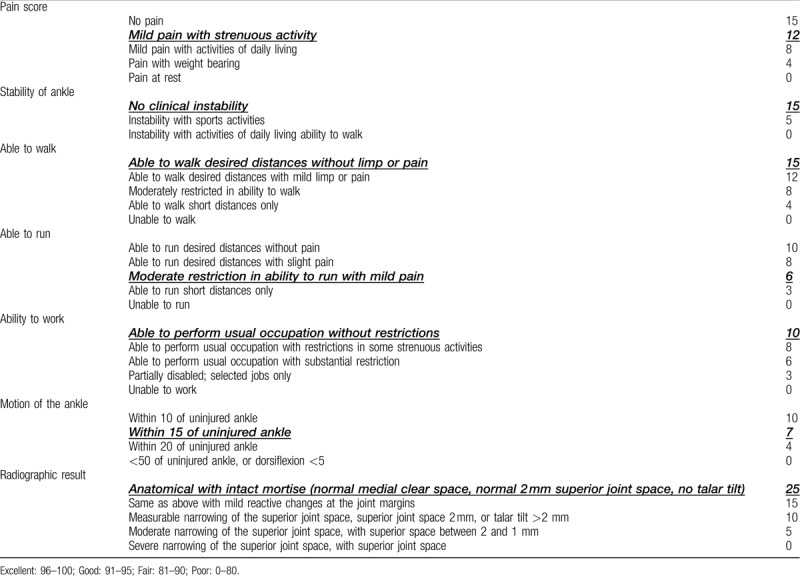
Baird and Jackson scoring system of this patient.

**Table 2:**
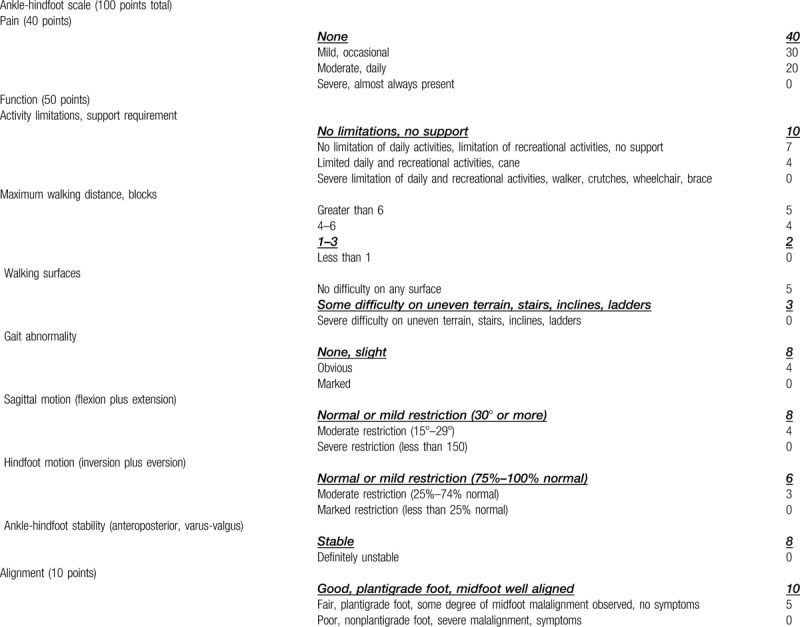
American orthopedic foot and ankle society ankle-hindfoot scale of this patient.

## 4 Discussion

The combination of a sagittal plane fracture of the talar neck and body with malleolar and calcaneal fractures is a rare diagnosis. In a case report of displaced vertical fracture of the talar neck extending through the body with vertical fracture of the medial malleolus and medial talar shift, the mechanism of injury was plantar hyperflexion, internal rotation, and axial compression.^[10]^ In another case in which the talar body in the sagittal plane was associated with a vertical fracture of the medial malleolus and fracture of the lateral process of the talus, talar fracture was fixed with 2 headless cancellous screws.^[2]^ Talar fractures are frequently fixed with 2 headless cancellous screws^[2,10,11]^; however, 1 headless screw with adequate splinting/casting with proper reduction may be sufficient.

Contact of the subtalar joint with dorsal and medial or varus displacement causes the greatest changes, indicating the need for multiple screw fixation.^[12]^

Lag screws can be used, unless there is significant neck comminution that would result in neck shortening or malalignment when the fracture is compressed.^[4]^

In the present case, talus fracture was a Hawkins classification type I, which has a 0% to 13% rate of osteonecrosis and a 0% to 10% rate of malunion.^[4]^ Decreased compression on the talar surface lessens vascular disruption due to the extensive surface vascular network.^[13]^ Fortunately, the outpatient department record and examination did not indicate AVN. We chose single headless screw fixation, which has a decreased risk of joint impingement if AVN was followed by the collapse of the talus bony surface and has the compression effect of a lag screw.

Osteoarthrosis is another common complication. Sneppen et al reported osteoarthritis as being more frequent in type C (sagittal shearing) than type D (posterior tubercle) fractures.^[14]^ We found only 1 study that reported single lag screw fixation.^[4]^ Fixation usually includes a double screw for compression and anti-rotation. As the talus is surrounded by joint capsules, ligaments, and synovial tissues and grossly appears as a square shape on coronary view, rotational force might not be as strong as that in other joints. If joint congruity can be restored and reduction can be performed with a single screw, the second screw might not be necessary.

Treatment of talar neck and body fracture with malleolar fracture and calcaneal fracture is challenging given its rarity and high complication rate. Anatomic reduction and rigid fixation are essential to the prognosis. As the Hawkins sign is clear on our case, single screw fixation for multi-fragment talar fracture is enough.

Essentially, this is our first time to perform single screw fixation for comminuted talar neck fracture. The technique is economical because less implant was used and particularly addresses concerns of patients worried about medical expenses. However, whether this surgical technique can provide uniform treatment for talar fracture with malleolar fracture or can be used in selected cases remains to be investigated.

## Acknowledgments

The authors are grateful to the patient, who gave his informed consent for publication. We would like to thank Editage (www.editage.com) for English language editing.

## Author contributions

**Conceptualization:** Kuan-Ju Chen, Hsien-Te Chen, Horng-Chaung Hsu.

**Data curation:** Chih-Yuan Ko, Tsung-Yu Ho.

**Formal analysis:** Chih-Hung Hung.

**Investigation:** Chih-Hung Hung.

**Methodology:** Chih-Yuan Ko, Chih-Hung Hung.

**Project administration:** Kuan-Ju Chen.

**Software:** Tsung-Yu Ho.

**Validation:** Chih-Hung Hung.

**Visualization:** Chih-Hung Hung.

**Writing – original draft:** Kuan-Ju Chen.

**Writing – review & editing:** Kuan-Ju Chen, Chih-Yuan Ko, Chih-Hung Hung.
